# Prediction of gestational diabetes mellitus in the first trimester: comparison of maternal fetuin-A, N-terminal proatrial natriuretic peptide, high-sensitivity C-reactive protein, and fasting glucose levels

**DOI:** 10.20945/2359-3997000000126

**Published:** 2019-04-15

**Authors:** Hatice Kansu-Celik, A. Seval Ozgu-Erdinc, Burcu Kisa, Rahime Bedir Findik, Canan Yilmaz, Yasemin Tasci

**Affiliations:** 1 University of Health Sciences Zekai Tahir Burak Health Practice Research Center Ankara Turkey University of Health Sciences, Zekai Tahir Burak Health Practice Research Center, Ankara, Turkey; 2 Gazi University Faculty of Medicine Department of Medical Biochemistry Ankara Turkey Gazi University Faculty of Medicine, Department of Medical Biochemistry, Ankara, Turkey

**Keywords:** Gestational diabetes, first trimester screening, fetuin-A, N-terminal peptide of proatrial natriuretic peptide, high sensitivity C-reactive protein, fasting plasma glucose

## Abstract

**Objective:**

We investigated the utility of maternal fetuin-A, N-terminal proatrial natriuretic peptide (pro-ANP), high-sensitivity C-reactive protein (hs-CRP), and fasting glucose levels at 11-14 gestation weeks for predicting pregnancies complicated by gestational diabetes mellitus (GDM).

**Subjects and methods:**

This prospective cohort study included 327 low-risk pregnant women who completed antenatal follow-up at a tertiary research hospital between January and April 2014. Maternal blood samples were collected between 11–14 gestational weeks in the first trimester of pregnancy and then stored at –80 °C until further analyses. During follow-up, 29 (8.8%) women developed GDM. The study population was compared 1:2 with age- and body mass index-matched pregnant women who did not develop GDM (n = 59). Fasting plasma glucose (FPG) levels and serum fetuin-A, pro-ANP, and hs-CRP levels were measured using automated immunoassay systems.

**Results:**

There was a significant negative correlation between fetuin-A and hs-CRP (CC = –0.21, p = 0.047) and a positive correlation between FPG and hs-CRP (CC = 0.251, p = 0.018). The areas under the receiver operating characteristic curve for diagnosing GDM were 0.337 (p = 0.013), 0.702 (p = 0.002), and 0.738 (p < 0.001) for fetuin-A, hs-CRP, and FPG, respectively. The optimal cut-off values were > 4.65, < 166, and > 88.5 mg/dL for maternal hs-CRP, fetuin-A, and FPG, respectively.

**Conclusion:**

Reduced fetuin-A, elevated hs-CRP, and FPG levels in women in the first trimester can be used for the early detection of GDM. Further research is needed before accepting these biomarkers as valid screening tests for GDM.

## INTRODUCTION

Fetuin-A is a novel biomarker that is mainly excreted from liver and adipose tissue and is associated with insulin resistance and metabolic syndrome (1). It increases insulin resistance by inhibiting the insulin receptor tyrosine kinase and by mediating toll-like receptor 4 signaling (2). Its secretion may differ in various disease states, and decreased levels of fetuin-A indicate its anti-inflammatory or protective effects as a negative acute-phase reactant (APR) (3). Sindhu and cols. demonstrated a negative correlation between plasma levels of fetuin-A and systemic inflammatory markers in patients with type 2 diabetes (4). Serum levels of fetuin-A increase mid-trimester in normal pregnancies (5). Only three previous studies have analyzed the potential relationship between fetuin-A levels and parameters of insulin resistance through the pregnancies complicated by GDM and normal pregnancies in the second trimester (6-8).

Atrial natriuretic peptide (ANP) is a peptide hormone mainly secreted by the heart. Although its main function is the regulation of hemodynamic homeostasis, several studies have observed a relationship between plasma glucose, insulin, and ANP levels (9). ANP circulates mainly as a 28-amino acid polypeptide, 99-126 amino acids at the C-terminal end prohormone, pro-ANP (10). A rapid increase in ANP levels occurs in response to acute hyperglycemia, and it is associated with poor glycemic control (11). Yuksel and cols. demonstrated that ANP levels are significantly reduced in patients with GDM beyond 26 weeks of gestation and negatively correlated with some parameters of insulin resistance (12).

High-sensitivity C-reactive protein (hs-CRP) is an inflammatory agent secreted by the liver. It may be correlated with obesity and insulin resistance (13). Increased maternal levels of hs-CRP in the first trimester are linked to increased risk of developing GDM (14).

To the best of our knowledge, this is the first study to investigate the utility of fetuin-A and pro-ANP levels in the first trimester as biomarkers for predicting GDM. We identified biochemical markers (fetuin-A, pro-ANP, hs-CRP, and FPG) related to GDM in the Turkish population in the first trimester to predict subsequent development of GDM.

## SUBJECTS AND METHODS

This prospective cohort study included pregnant women who attended their routine antenatal follow-up at 11 and 14 gestational weeks at the Zekai Tahir Burak Women’s and Research Hospital between January and April 2014. This study was conducted in accordance with the Declaration of Helsinki (15). The institutional review board (23# 26/12/2013) approved the study. Exclusion criteria included age < 18 years or > 40 years, diagnosis of chronic disease before conception (diabetes, hypertension, thyroid dysfunction, uncontrolled endocrine illness, or abnormal renal function), FPG levels exceeding 126 mg/dL, or postprandial 2^nd^ h glucose level or glucose challenge test (GCT) value exceeding 200 mg/dL at gestational weeks of 24-28, history of a positive glucose tolerance test in the first trimester, and having had a previous pregnancy. Medical history and demographic characteristics including age, parity, pre-pregnancy body mass index (BMI), and smoking were noted. Gestational age was estimated via ultrasonography results between 11 and 14 weeks of gestation. BMI was calculated as weight (kg)/height (m)^2^. Type of delivery, complications during gestation, and neonatal outcomes were recorded. All subjects underwent a two-step GDM screening between 24 and 28 weeks of gestation A positive 50 g GCT was defined as a glucose level of at least 140 mg/dL 1 h after the glucose challenge. In case of positive 50g GCT, 100 g oral glucose tolerance test (OGTT) was performed following 3 days of normal diet. GDM was diagnosed if there were two or more abnormal values on a 100g OGTT performed according to the criteria identified by Carpenter and Coustan (FPG: 95 mg/dL, 1^st^ h: 180 mg/dL, 2^nd^ h: 155 mg/dL, 3^rd^ h: 140 mg/dL) (16). Healthy controls were defined as pregnant women with a negative 50 g GCT (1^st^ h < 140 mg/dL). The control group was selected and recruited for the study using a simple random-sampling method, and 59 pregnant women who did not develop GDM were matched by age and BMI at a 1:2 ratio.

Venous blood samples for fetuin-A, pro-ANP, hs-CRP, and FPG were collected at the routine obstetric examination at 11-14 weeks of gestation. Maternal blood samples were immediately centrifuged, and serum was separated and stored at –80 °C until use. All of the laboratory measurements were performed simultaneously in the same laboratory by the same technician.

### Biochemical assays

Serum levels of fetuin-A and pro-ANP in separate samples were quantified using enzyme linked immunosorbent assay (ELISA) kits according to the manufacturer’s protocols (Cat.No:YHB1184Hu, Lot No: 20160901, and Cat.No:YHB2435Hu, Lot No: 20160901; YEHUA Biological Technology, Shanghai, China). These kits use ELISAs based on a biotin double antibody sandwich technology to assay fetuin-A and pro-ANP. The detection limits of the assays were between 10-4,000 mg/L and 5-2,000 μmol/L, respectively, and the intra- and inter-assay coefficients of variation were < 10 and < 12%, respectively.

Serum levels of glucose were measured using a Beckman AU5800 biochemistry auto-analyzer with the hexokinase method. The total coefficient of variation was < 2.0%, and the reference interval was 60-100 mg/dL. Serum levels of hs-CRP were measured using a Siemens BN ProSpec nephelometer. The intra- and inter-assay coefficients of variation were 4.0% and 4.6%, respectively. The reference interval was < 2.87 mg/L.

### Statistical analyses

To calculate statistical power, we accepted the GDM prevalence in our population as 5% and the effect size as 0.8 (17). The sample size calculation for this study population of 88 women with an allocation ratio of 2 and a two-tailed sample comparison with a 5% level of significance (alpha) had a power of 0.94. This sample size was sufficient to detect a 0.5 standard deviation difference in continuous variables given the same power and significance level. Sample size calculations were performed using the G*Power v.3.1.5 general power analysis program (18).

The mean and standard deviation (SD) were calculated for continuous variables, and categorical variables are expressed as numbers and percentages. Chi-square tests or Fisher’s exact tests were used to compare categorical variables. The normality of variables was analyzed using the Kolmogorov-Smirnov test. Independent sample *t*-tests were used to compare continuous variables with normal distribution. The Mann–Whitney U test was used to analyze non-normally distributed data. Correlation analyses were done using Spearman’s coefficient. Pearson’s test was performed when necessary. The accuracy of each test was evaluated separately, and a multiple binary logistic regression model was generated to identify variables that were significantly associated with the outcome of interest. For multivariate analyses, possible factors identified in univariate analyses were further analyzed by binary logistic regression to determine independent predictors of GDM in the first trimester of pregnancy. The Hosmer–Lemeshow goodness of fit statistic was used to assess model fit. A 5% type-1 error level was used to infer statistical significance. The sensitivity, specificity, positive predictive value (PPV), negative predictive value (NPV), positive likelihood ratio (PLR), negative likelihood ratio (NLR), and diagnostic odds ratio (OR) with their associated 95% confidence intervals (CIs) were determined for each method. The detection and false-positive rates in the prediction of GDM were estimated using receiver operating characteristic (ROC) curves, and the diagnostic power of these screening tests to predict GDM in early pregnancy was assessed by comparing the areas under the ROC curve (AUROCs). Youden’s index was used to select the cut-off value for diagnosis of GDM for each test (19). A two-tailed *p*-value < 0.05 was considered statistically significant. The statistical software package IBM SPSS 21.0 (IBM Corp., Armonk, NY, USA) was used for data analyses.

## RESULTS

During the study period, 350 healthy low-risk pregnant women at 11-14 gestational weeks met the inclusion criteria. A total of 23 patients were excluded due to loss to follow-up, not participating in GDM screening, or having a chronic disease before pregnancy. In the final analysis, a total of 327 women completed antenatal follow-up in our hospital, 29 of whom (8.8%) developed GDM, and 59 healthy controls were age- and BMI-matched in a 1:2 ratio.

The demographic characteristics and obstetric and neonatal outcomes of the patients are shown in Table 1. There was no statistically significant difference between groups in terms of age, gravidity, parity, pre-pregnancy BMI, smoking status, gestational age at delivery, type of delivery, birth weight, Apgar scores at first and fifth minute, and NICU requirement (*p* > 0.05).

**Table 1:** Demographics, obstetric and neonatal outcomes

Variable	GDM Group (n = 29)	Control groups (n = 59)	*p*
Age (years) (Mean ± SD)	29.96 ± 3.79	29.28 ± 3.69	0.121
Gravidity Median (Min-Max)	3 (1-8)	2 (1-5)	0.139
Parity (Mean ± SD) (Min-Max)	1 (0-3)	1 (0-3)	0.11
Pre-pregnancy BMI (Mean ± SD)	27.74 ± 2.14	27.41 ± 2.18	0.656
Smoking n (%)	1 (34%)	6 (10%)	0.418
Gestational age at delivery (week) (Mean ± SD)	38.60 ± 1.82	38.96 ± 1.56	0.359
Mode of delivery n (%)			0.656
Vaginally	22 (76%)	47 (80%)	
C-section	7 (24%)	12 (20%)	
Birthweight (g) (Mean ± SD)	3391 ± 455	3372 ± 409	0.85
Apgar scores Median (Min-Max)			
1th min	8 (6-9)	8 (6-9)	0.321
5th min	10 (8-10)	10 (8-10)	0.234
NICU (n,%)	3 (10%)	5 (8.4%)	0.115

*p < 0.05: significant; BMI: body mass index; NICU: requirement of neonatal intensive care unit.

Fetuin-A levels were significantly lower, and hs-CRP levels were significantly higher, in cases of complicated GDM compared to uncomplicated ones (154 [95% CI: 47-2777] ng/mL vs. 210 [44-1860] ng/mL, *p* = 0.013; and 7.95 [3.14–27.7] ng/mL vs. 4.63 [3.11-24.2] ng/mL, *p* = 0.032, respectively). There was no statistically significant difference between groups in terms of pro-ANP levels (*P* < 0.05) (Table 2).

**Table 2:** Maternal fetuin-A, pro-ANP, and hs-CRP levels between two groups

Variable	GDM Group (n = 29)	Control groups (n = 59)	*p*
Fetuin-a (ng/mL)	154 (47-2777)	210 (44-1860)	0.013*
Pro-ANP (mg/dL)	694 (461-4957)	721 (484-4551)	0.144
Hs-CRP (ng/mL)	8.89 (3.14-27.7)	4.63 (3.11-24.2)	0.003*
FPG (mg/dL)	92.93 ± 7.84	85.79 ± 8.17	<0.001*

*p < 0.05, significant. BMI: body mass index; Pro-ANP: N-terminal peptide of proatrial natriuretic peptide; hs-CRP: high sensitive C-reactive protein; FPG: fasting plasma glucose.

According to Spearman rank correlation analyses, there was a significant inverse correlation between fetuin-A and hs-CRP (CC = –0.93, *p* = 0.009), and a positive correlation between hs-CRP and FPG, but no correlation between fetuin-A and FPG levels (CC = –0.186, *p* = 0.083).

**Figure 1:**
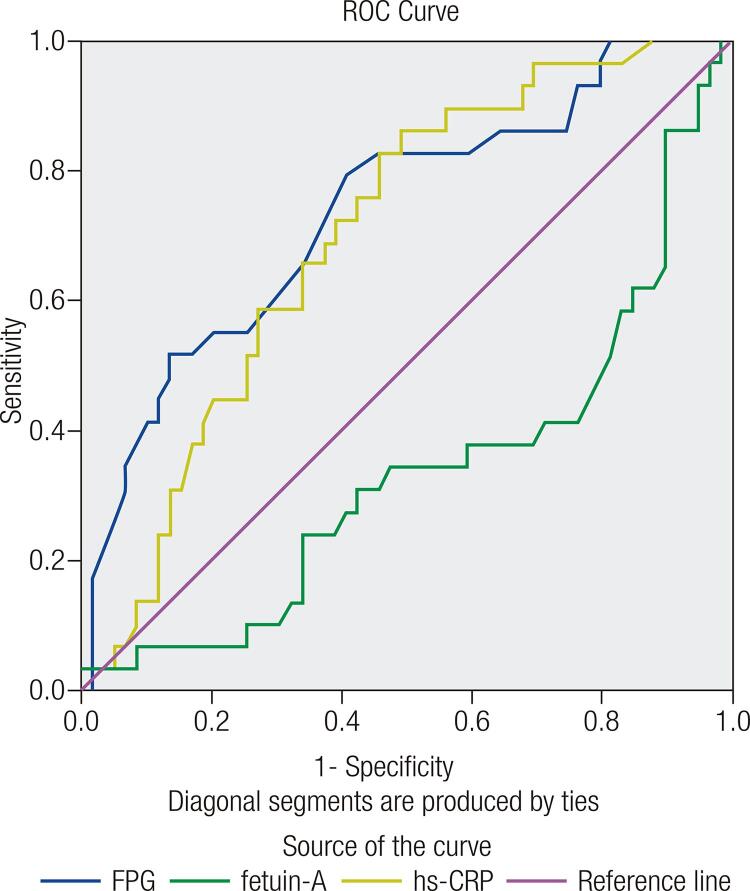
Area under receiver operating characteristic (ROC) curves for FPG, fetuin-A and hs-CRP in predicting GDM.

The area under ROC curves of fetuin-A, hs-CRP, and FPG for diagnosing GDM were 0.337 (95% CI: 0.212--0.461, *p* = 0.013), 0.702 (95% CI: 0.592-0.812, *p* = 0.002), and 0.738 (95% CI: 0.626-0.850, *p* < 0.001), respectively (Figure 1). Table 3 shows the different measurements of diagnostic accuracy of FPG, hs-CRP, and fetuin-A. Maternal hs-CRP levels above 4.65 ng/mL had the highest sensitivity (86.21%, 95% C1: 67.43-95.49), NPV (88.24%, 95% CI: 71.61–96.16), and PLR (61.36%, 95% CI: 50.35-71.38), with a diagnostic accuracy of 88.64%. Fetuin-A levels below 166 ng/mL had the highest specificity (76.27%, 95% CI: 63.11-85.98), NLR (64.77, 95% CI: 53.79-74.45), and PPV (54.84%, 95% CI: 36.3-72.22) with a diagnostic accuracy of 70.45%. FPG levels above 88.5 mg/dL had a sensitivity of 79.31% (95% C1: 59.74-91.29), specificity of 59.32% (95% CI: 45.76-71.67), and the PPV and NPV were 48.94% (95% CI: 34.3-63.74) and 85.37% (95% CI: 70.14-93.91), respectively, with a diagnostic accuracy of 85.23%.

**Table 3:** Diagnostic performances of first trimester FPG, hs-CRP and fetuin-A

	FPG	hs-CRP	fetuin-A
Sensitivity (%) and 95 CI (%)	79.31 (59.74-91.29)	86.21 (67.43-95.49)	58.62 (39.13-75.91)
Specificity (%) and 95 CI (%)	59.32 (45.76-71.67)	50.85 (37.64-63.95	76.27 (63.11-85.98)
Positive Likelihood Ratio and 95 CI (%)	53.41 (42.51-64.01)	61.36 (50.35-71.38)	35.23 (25.55-46.21)
Negative Likelihood Ratio and 95 CI (%)	46.59 (35.99-57.49)	38.64 (28.62-49.65)	64.77 (53.79-74.45)
Positive Predictive Value (%) and 95 CI (%)	48.94 (34.3-63.74)	46.3 (32.84-60.27)	54.84 (36.3-72.22)
Negative Predictive Value (%) and 95 CI (%)	85.37 (70.14-93.91)	88.24 (71.61-96.16)	78.95 (65.75-88.2)
Diagnostic Odds Ratio and 95 CI (%)	5.59 (1.98-15.78)	6.47 (2.0-20.88)	4.55 (1.76-11.8)
**Diagnostic Accuracy (%)**	**85.23**	**88.64**	**70.45**

## DISCUSSION

We evaluated the diagnostic value of first trimester fetuin-A, pro-ANP, and hs-CRP levels in predicting GDM. Low levels of fetuin-A and high levels of hs-CRP at 11 to 14 weeks of gestation were associated with GDM later in pregnancy. In addition, there was an inverse correlation between fetuin-A and hs-CRP levels.

GDM affects 8%-18% of all pregnancies, and with impaired glucose tolerance first recognized during the second or third trimester of pregnancy, it is a main cause of maternal and neonatal morbidity and mortality (20). The cornerstone of management is glycemic control, and poor control during pregnancy has been associated with miscarriage, preterm birth, stillbirth, macrosomia, urinary tract infection, polyhydramnios, shoulder dystocia, operative delivery, neonatal hyperbilirubinemia-hypocalcemia, and increased NICU admission (21).

Therefore, some first-trimester markers may help to predict this complication and improve the management of such cases (22-24). Recent studies have found changes in the expression of fetuin-A, pro-ANP, and hs-CRP in pregnant women with GDM (12,14,25,26).

Fetuin-A, a member of the cystatin superfamily of protease inhibitors, is secreted by the liver in adults and by various fetal tissues (27). It is associated with insulin resistance and metabolic syndrome. It binds to the β-subunit of the insulin receptor, thus activating insulin receptor kinase (2). Some studies have reported high concentrations of fetuin-A in patients with type 2 DM, and others have reported low concentrations (25,28,29); therefore, the results have been conflicting and limited. Farhan and cols. observed no differences in fetuin-A levels in the third trimester of pregnancy and in the postpartum period in a group of GDM patients (6), whereas Kalabay and cols. and Iyidir and cols. found increased levels of fetuin-A in women with GDM compared to healthy pregnant women at 20-40 gestational weeks, and between 24-28 gestational weeks, respectively (7,8). In healthy individuals, fetuin-A concentrations range from 450 to 600 μg/mL (30). In Kalabay and cols., fetuin-A concentrations increased with gestational age in healthy pregnant women, but interval values during the pregnancy period were not clearly reported by the authors (7). Iyidir and cols. observed a mean level of 35 ng/dL in a GDM group at gestational weeks 24 and 28 (8). In Briana and cols., the mean concentration in healthy pregnant women during the first stage of labor or before elective C-section was 43 ng/dL (31). To the best of our knowledge, there are no data evaluating the utility of maternal fetuin-A levels at 11-14 weeks of gestation for predicting GDM. In the current study, we found that fetuin-A levels were significantly lower in women with GDM than in healthy pregnant women. Low levels of fetuin-A can be explained by several possible mechanisms. Some studies have reported that low concentrations of fetuin-A are related to inflammation and vascular calcifications, whereas high levels are associated with dyslipidemia and metabolic syndrome (1). Subclinical inflammation in the early period of pregnancy may lead to a decrease in fetuin-A levels due to its protective effects as an APR (3). In addition, the first trimester of pregnancy is known as the insulin-sensitive period. Catalano and cols. reported that insulin resistance progressively increases during the second trimester of pregnancy (32). We found no correlation between fetuin-A levels and FPG, although FPG was significantly higher in patients who subsequently developed GDM. Future longitudinal studies showing changes in fetuin-A levels over time in women developing GDM are needed.

Recent cross-sectional studies have demonstrated an inverse correlation between ANP and metabolic syndrome, insulin resistance, and FPG. Magnusson and cols. demonstrated that low ANP concentrations predict later development of diabetes, and suggested that ANP deficiency may have a pivotal role in diabetes development (33). Yuksel and cols. demonstrated that ANP levels were significantly lower in patients with GDM beyond 26 weeks of gestation and negatively correlated with some parameters of insulin resistance (12). This may be because low levels of ANP may become evident due to either increased clearance or decreased cardiac production of ANP in subsequent gestational weeks (34). In our study, pro-ANP levels were lower in women with GDM than in healthy pregnant women, but the difference was not statistically significant. A progressive decline in ANP may lead to insulin resistance in the second trimester.

GDM is associated with both short- and long-term risks for mothers and fetuses during pregnancy and postpartum (35). Many validated prediction models are being developed to recognize GMD and intervene in the early weeks of pregnancy (35,36). Among these markers, hs-CRP levels in the first trimester are linked to an increased risk of developing GDM, with high specificity and diagnostic odds ratio (14,37). High hs-CRP values are indicative of increased inflammation at baseline, which is also an independent risk factor for developing GDM (37). Consistent with previous studies, we demonstrated a positive association between hs-CRP levels and GDM prediction. On the other hand, hs-CRP is a positive APR. We found that levels of fetuin-A, a negative APR, were significantly lower in women with GDM than in healthy pregnant women. Previous studies have documented that fetuin-A levels are inversely correlated with CRP levels in serum (38). Our findings are consistent with the literature. We also demonstrated that hs-CRP has better diagnostic accuracy for GDM than fetuin-A and FPG (88.64 vs. 70.45 and 85.23%, respectively).

Similar to previous studies, we detected higher first trimester FPG levels among women who subsequently developed GDM. Riskin-Mashiah and cols. and Ozgu-Erdinc and cols. reported FPG cut-off values of 80-85 mg/dL (75-55% sensitivity and 52-75% specificity) and 90 mg/dL (55.1% sensitivity and 71% specificity), respectively for predicting GDM (14,39). We found a similar result of 88.5 mg/dL, with 79.3% sensitivity and 59.3% specificity. FPG has a test validity comparable to that of hs-CRP but is much easier and less costly to determine. Thus, for clinicians, this is the most valuable and helpful result of our study.

In the management of GDM, treatment modalities aimed to improve insulin sensitivity may be useful. Controlling weight gain during pregnancy reduces the incidence of GDM in pregnant women (40). Exercise improves glycemic control, and exercising both prior to and during pregnancy has the greatest correlation with protection against GDM development. Changes in diet, exercise, and achieving desirable gestational weight gain should be encouraged to improve insulin sensitivity (40).

We did not include non-pregnant individuals as a control group. This is a limitation for our study. Despite this, our study is the first trial investigating the circulating fetuin-A and pro-ANP levels in the first trimester of pregnancy, and our results provide important information on the associations between fetuin-A, hs-CRP, and FPG and subsequent development of GDM.

Elevated hs-CRP and FPG and reduced fetuin-A levels (compared to levels in uncomplicated pregnancies) at 11 to 14 weeks of gestation are associated with subsequent development of GDM. Thus, these markers may be useful for early detection and intervention in GDM. However, further studies with larger sample sizes are needed before accepting these markers as valid screening tests for GDM.
